# Genotype‐phenotype correlations in multiple lesions of familial cerebral cavernous malformations concerning phosphatidylinositol 3‐kinase catalytic subunit alpha mutations

**DOI:** 10.1002/ctm2.1610

**Published:** 2024-03-07

**Authors:** Jie Wang, Jihong Tang, Yingxi Yang, Yuming Jiao, Ran Huo, Hongyuan Xu, Shaozhi Zhao, Yingfan Sun, Qiheng He, Qifeng Yu, Shuo Wang, Jizong Zhao, Jiguang Wang, Yong Cao

**Affiliations:** ^1^ Department of Neurosurgery Beijing Tiantan Hospital, Capital Medical University Beijing China; ^2^ China National Clinical Research Center for Neurological Diseases Beijing China; ^3^ SIAT‐HKUST Joint Laboratory of Cell Evolution and Digital Health, Shenzhen‐Hong Kong Collaborative Innovation Research Institute Shenzhen China; ^4^ Division of Life Science, Department of Chemical and Biological Engineering, and State Key Laboratory of Molecular Neuroscience The Hong Kong University of Science and Technology Kowloon China; ^5^ Hong Kong Center for Neurodegenerative Diseases Hong Kong SAR China; ^6^ Beijing Neurosurgical Institute, Capital Medical University Beijing China

Dear Editor,

Our study suggested that in familial multiple cerebral cavernous malformations (CCMs), symptomatic intracerebral haemorrhage (ICH) lesions and dot‐sized lesions harbour distinct clinical features and genotypes. Bevacizumab, a vascular endothelial growth factor (VEGF) inhibitor, might be a potential therapeutic agent for familial CCMs.

CCMs affect approximately 0.16% to 0.8% of the general population, which is a major cause of ICH in young adults. Sporadic cases often occur as a single lesion without a family history, whereas familial cases are characterized by multiple lesions with a positive family history.^1^ Based on magnetic resonance imaging (MRI), two subtypes of lesions of familial CCMs were found: ICH lesions exhibit subacute haematoma, while dot‐sized lesions show haemosiderin staining.^2^ The genotype‐phenotype correlations of sporadic CCMs are well studied. However, the phenotype, genotype and relationship of these multiple lesions in familial CCMs remain unclear.

To clarify the clinical features of the two subtype lesions, 10 familial CCM patients were enrolled (Table S1). Among 10 patients, there were 12 ICH lesions and 505 dot‐sized lesions (Figure 1A,B), and the pattern of these lesions both distributed throughout the brain parenchyma.^3^, ^4^ After a follow‐up time of 3230.7 lesion‐years, haemorrhage events occurred in 11 lesions and Kaplan‐Meier analysis revealed that the annual incidence rate for haemorrhage events of ICH lesions (22.0 per 100 lesion‐years) was significantly higher than that of dot‐sized lesions (0.03 per 100 lesion‐years) (*p* < 0.001) (Figure 1C–E).

**FIGURE 1:**
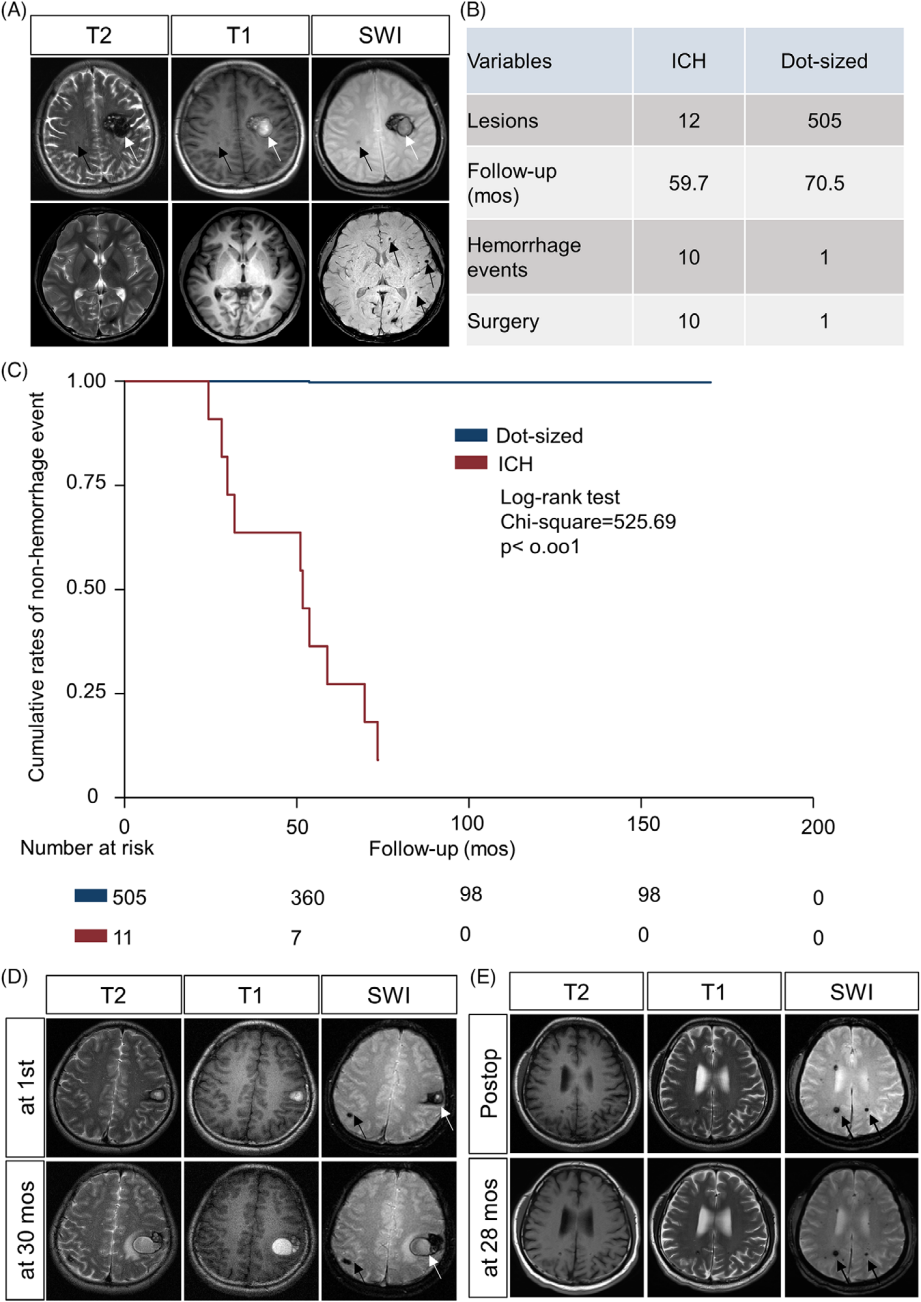
Symptomatic intracerebral haemorrhage (ICH) lesions and dot‐sized lesions in familial cerebral cavernous malformations (CCMs) have distinct natural courses. (A) The classification of multiple lesions in familial CCMs. White arrows (ICH lesions) and black arrows (dot‐sized lesions). (B) Total lesions, follow‐up months, haemorrhage event and surgical resection of enrolled patients. A patient with two ICH lesions was resected a lesion at first hospitalization. (C) The Kaplan‐Meier curve showing the cumulative rate of haemorrhage events between ICH lesions and dot‐sized lesions (black arrows) (Log‐rank test *p* < .001). (D) Representative follow‐up magnetic resonance imaging (MRI) of ICH lesions (white arrows) and dot‐sized lesions (black arrows). (E) Representative follow‐up MRI of dot‐sized lesions (black arrows) after surgical resection of ICH lesion. Postop, postoperative.

To investigate the genotypes of ICH and dot‐sized lesions, DNA sequencing was performed on the surgical samples from familial CCMs. First, we investigated the genotypes between pairings of symptomatic ICH lesions and their adjacent independent dot‐sized lesions (Figure S1). Combining whole exome sequencing (WES) and droplet digital polymerase chain reaction (ddPCR), 3 ICH lesions harboured CCM germline mutations and phosphatidylinositol 3‐kinase catalytic subunit alpha (*PIK3CA*) somatic mutations, whereas three dot‐sized lesions only had CCM germline mutations (Figure 2A and Table S2). Furthermore, through WES analysis of 12 ICH formalin‐fixed paraffin‐embedded (FFPE) samples from 10 patients enrolled in imaging follow‐up (Figure S1), CCM germline mutations were detected (Tables S1 and S2). Because the total DNA extracted from FFPE samples was limited, ddPCR was applied to detect the prevalent *PIK3CA* mutations.^5^ *PIK3CA* mutations were found in 10 samples (Figure 2A,B, Figures S2–S6 and Table S3). Overall, 86.7% (13/15) ICH lesions harboured somatic *PIK3CA* plus CCM germline mutations, whereas 100% (3/3) dot‐sized lesions had only CCM germline mutations (*p* = 0.012) (Figure 2C, Figure S2–S6 and Table S3). Additionally, three (16.7%) specimens were detected with somatic CCM mutations by WES (Table S4). In familiar cases, CCM germline mutations occur earlier than somatic *PIK3CA* mutations, nevertheless, CCM mutations may be a secondary event following the mutation of PI3K pathway genes in sporadic cases.^3^, ^4^, ^5^

**FIGURE 2:**
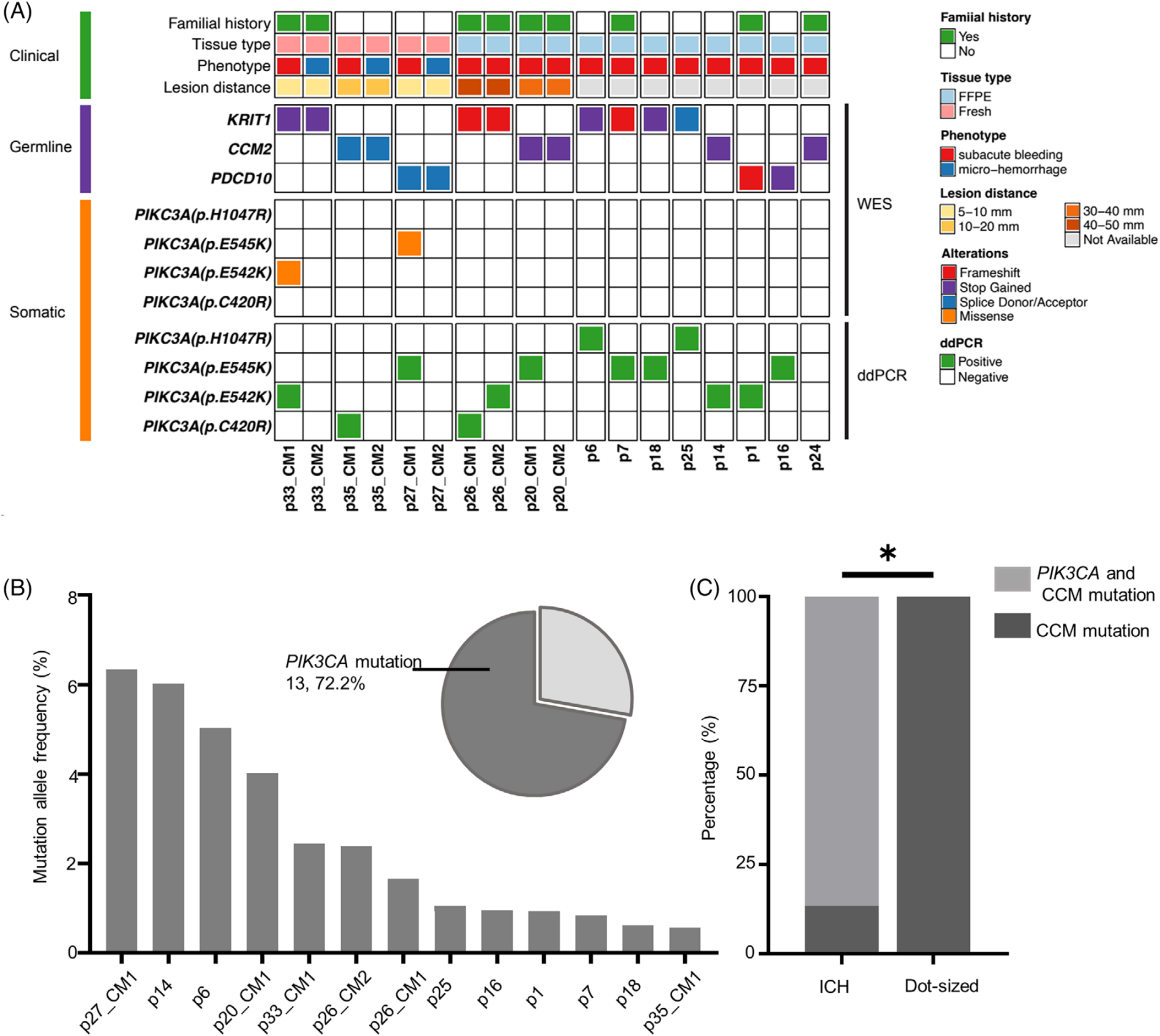
Mutational landscape of familial cerebral cavernous malformations (CCMs). (A) Summary of germline and somatic mutations in 18 two subgroup lesions of CCM (three dot‐sized lesions and 15 intracerebral haemorrhage [ICH] lesions) by whole exome sequencing (WES) and/or droplet digital polymerase chain reaction (ddPCR). (B) ddPCR showed ranked mutation allele frequencies of phosphatidylinositol 3‐kinase catalytic subunit alpha (*PIK3CA*) mutation in the study. Mutational incidence of *PIK3CA* mutation detected by ddPCR was displayed by a pie chart. (C) Enrichment of genotype in ICH and dot‐sized lesions. *, *p* < .05.

Two subgroups’ lesions harboured different genotypes and related phenotypes, which implies an underlying molecular mechanism within them. Recent studies indicated that *PIK3CA* mutation was associated with haemorrhagic events in CCMs.^6^, ^7^, ^8^ To confirm the mechanism of *PIK3CA* mutation in familial CCMs, we activated PI3K signalling in human umbilical vein endothelial cells (HUVECs) by transfecting with short‐interfering *PTEN* (si*PTEN*). Compared to the control group, the levels of phosphorylated AKT (p‐AKT), VEGF A (VEGFA), phosphorylated rho‐associated kinase 2 (p‐ROCK2) and thrombomodulin (TM) in the *PTEN*‐knockdown HUVECs were elevated. The TM expression was reversed by MK‐2206 (PI3K inhibitor) and fasudil (ROCK inhibitor) (Figure 3A). Furthermore, we investigated the effect of PI3K pathway activation in *Krit1*‐knockdown HUVECs (Figure S7). We found that in *Krit1*‐knockdown HUVECs, PI3K pathway activation also increased the expression of p‐AKT, VEGFA, p‐ROCK2 and TM (Figure 3A). TM expression was also reversed by MK‐2206 and fasudil.

**FIGURE 3:**
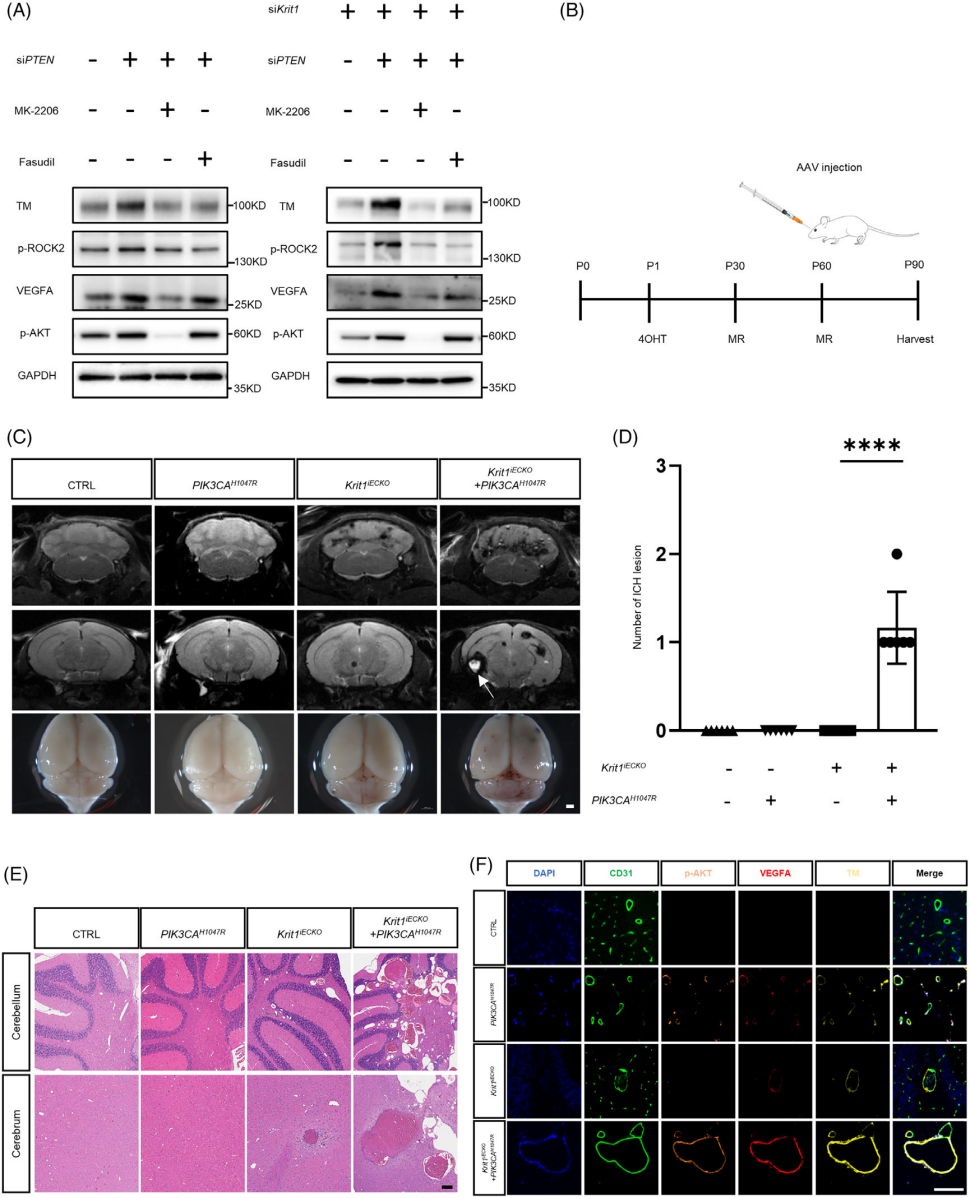
Endothelial PI3K activation promotes vascular endothelial growth factor (VEGF) signalling pathway resulting in aggravating cerebral cavernous malformation (CCM) haemorrhage in mice. (A) Western blot of the expression of phosphor‐AKT, VEGFA, ROCK2 and thrombomodulin (TM) in human umbilical vein endothelial cells (HUVECs) transfected with si*PTEN* (left panel) and HUVECs transfected with si*PTEN* and si*Krit1* (right panel), and the TM expression was reversed by MK‐2206 (an inhibitor of PI3K signalling) and Fasudil (an inhibitor of ROCK signalling). (B) Schematic diagram of the experimental approach. 4‐hydroxytamoxifen (4OHT) injection at P1 was used to induce deletion of *Krit1* (*Krit1* ^iECKO^) in the endothelium, and mice were performed 7 T magnetic resonance imaging (MRI) at P30 and P60, then adeno‐associated virus (AAV)‐control and AAV‐*PIK3CA* ^H1047R^ were administered in P60 mice by retro‐orbital venous sinus injection. Finally, the mice were performed MRI and harvested at P90. (C) Representative MRI and visual images of the four groups (CTRL: AAV‐control+*Krit1* ^fl/fl^, *PIK3CA* ^H1047R^: AAV‐*PIK3CA* ^H1047R^+*Krit1* ^fl/fl^, *Krit1* ^iECKO^: AAV‐control+ *Krit1* ^iECKO^, *PIK3CA* ^H1047R^+*Krit1* ^iECKO^: AAV‐*PIK3CA* ^H1047R^+*Krit1* ^iECKO^, each n = 6). White arrows show intracerebral haemorrhage (ICH) lesions in mice, bar, 1000 um. (D) Quantitation of ICH lesions on MRI among four groups at P90 (*n* = 6), ****, *p* < .0001. (E) Comparison of CCM lesions located in cerebrum and cerebellum by histologic examination in four groups (*n* = 6), bar = 200 um. (F) Expression of phosphor‐AKT, VEGFA and TM in the brain endothelial cells by immunofluorescence staining at P90, bar = 100 um.

We then validated the genotype‐phenotype correlations of the two lesion subgroups in vivo. C57BL/6 mice with endothelial cell‐specific deletion of *Krit1* (*Krit1* ^iECKO^) were induced by 4‐hydroxytamoxifen injection at postnatal day 1 (P1) as described previously (Figure S8).^9^ Then, adeno‐associated virus (AAV)‐control or AAV‐*PIK3CA* ^H1047R^ was administered to *Krit1* ^iECKO^ or *Krit1* ^fl/fl^ mice at P60 via retro‐orbital sinus injection. Brain MRI and histologic examination were performed at P90 (Figure 3B). On MRI, typical subacute ICH lesions were observed in the AAV‐*PIK3CA* ^H1047R^+*Krit1* ^iECKO^ group, while only chronic small lesions were observed in the AAV‐control*+Krit1* ^iECKO^ group (Figure 3C,D). The mean numbers of lesions in the AAV‐*PIK3CA* ^H1047R^+*Krit1* ^iECKO^ and AAV‐control*+Krit1* ^iECKO^ group were 98.2 and 44.5, respectively (Figure 3D and Figure S9A), and a higher number of larger lesions (> 10 000 µm^2^) were observed in the *PIK3CA* ^H1047R^+*Krit1* ^iECKO^ group than in the AAV‐control*+Krit1* ^iECKO^ group (45.0 vs. 11.0, Figure 3E and Figure S9A). Furthermore, compared with *Krit1* ^fl/fl^+AAV‐PIK3CA^H1047R^ or AAV‐control+*Krit1* ^iECKO^ mice, the brain endothelial cells of AAV‐*PIK3CA* ^H1047R^+*Krit1* ^iECKO^ mice showed elevated expression of VEGFA and TM (Figure 3F and Figure S9B).

Vitro and vivo experiments both indicated that PI3K‐VEGF pathway activation plays a vital role in the genotype‐phenotype correlations. We hypothesized that VEGF inhibitors might restrain bleeding. Bevacizumab, a VEGF inhibitor, is an effective therapy for cancers. To investigate the effect of bevacizumab, P60 *Krit1* ^iECKO^ mice were injected with AAV‐*PIK3CA* ^H1047R^ via retro‐orbital sinus and bevacizumab through the tail vein, and the control mice were injected with AAV‐*PIK3CA* ^H1047R^ and vehicle alone. Then, the mice were subjected to MRI and histological examination at P90 (Figure 4A). Surprisingly, bevacizumab treatment inhibited ICH formation (Figure 4B,C). Furthermore, haematoxylin and eosin staining revealed that bevacizumab dramatically decreased the number of CCM lesions and prevented the formation of larger CCMs (Figure 4D–F).

**FIGURE 4:**
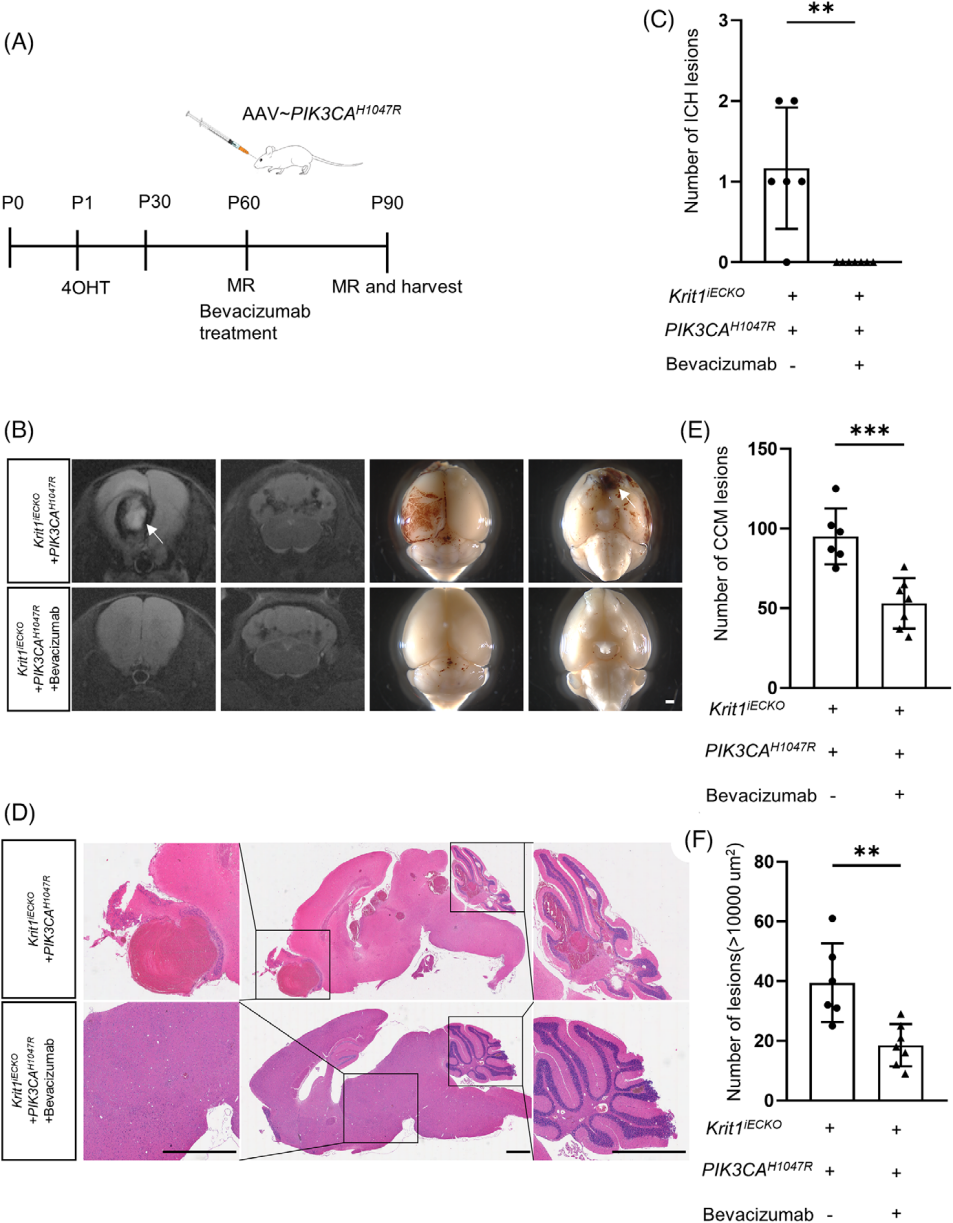
Bevacizumab prevents CCM formation in phosphatidylinositol 3‐kinase catalytic subunit alpha (*PIK3CA*)^H1047R^+*Krit1* ^iECKO^ mice. (A) Schematic diagram of the experimental approach. 4OHT injection at P1 was used to induce deletion of *Krit1* (*Krit1* ^iECKO^) in the endothelium of mice, and mice were performed magnetic resonance imaging (MRI) at P30 and P60, then adeno‐associated virus (AAV)‐*PIK3CA* ^H1047R^ were administered in P60 mice by retro‐orbital venous sinus injection, meanwhile, the mice were injected through the tail vein with either bevacizumab with a dose of 2 mg/kg or vehicle twice a week for total three weeks. Finally, the mice were performed MRI and harvested at P90. (B) Representative MRI and visual images of the two groups (AAV‐*PIK3CA* ^H1047R^+*Krit1* ^iECKO^+bevacizumab, n = 7, AAV‐*PIK3CA* ^H1047R^+*Krit1* ^iECKO^+PBS, n = 6). White arrows show intracerebral haemorrhage (ICH) lesions in mice, bar, 1000 um. (C) Quantitation of ICH lesions on MRI among two groups at P90(AAV‐*PIK3CA* ^H1047R^+*Krit1* ^iECKO^+ bevacizumab, *n* = 7, AAV‐*PIK3CA* ^H1047R^+*Krit1* ^iECKO^+PBS, *n* = 6), **, *p* < .01. (D) Comparison of CCM lesions located in cerebrum and cerebellum by histologic examination in two groups (AAV‐*PIK3CA* ^H1047R^+*Krit1* ^iECKO^+bevacizumab, *n* = 7, AAV‐*PIK3CA* ^H1047R^+*Krit1* ^iECKO^+PBS, *n* = 6), bar = 2000 um. (E, F) Quantitation of total CCM lesions and lesion sized more than 10000 um^2^ on histologic examination among two groups at P90 (AAV‐*PIK3CA* ^H1047R^+*Krit1* ^iECKO^+bevacizumab, *n* = 7, AAV‐*PIK3CA* ^H1047R^+*Krit1* ^iECKO^+PBS, *n* = 6). **, *p* < .01, ***, *p* < .001.

Our study has several limitations. First, for ethical considerations, we cannot obtain more surgical specimens of dot‐sized lesions in the familial CCMs; second, some paired blood samples were unavailable in FFPE samples and the mutation type of surgical samples (germline or somatic) was identified by minor allele frequency (MAF); finally, for the suboptimal quality of sample DNA, the MAF of CCM germline mutation were below 50%, particularly in the FFPE samples.

Here, we identified that in familiar CCMs, the two subgroup lesions have distinct genotype‐phenotype correlations concerning *PIK3CA* mutations, which implies differentiated management strategy might be taken. Bevacizumab, as a promising therapeutic agent for familial CCMs, requires further prospective clinical study to evaluate its safety and efficacy due to the risk of complications such as hypertension and cardiac ischemia.^10^

## AUTHOR CONTRIBUTIONS

Jiguang Wang and Yong Cao conceived and designed the study; Jie Wang contributed to clinical data collection, samples collection, vivo and vitro experiment, data analysis and wrote the manuscript; Jihong Tang and Yingxi Yang contributed to bioinformatics analysis; Ran Huo and Hongyuan Xu contributed to experimental design and implementation; Yingfan Sun, Yuming Jiao, Qiheng He, Shaozhi Zhao and Qifeng Yu helped with collecting the samples. Shuo Wang, Jizong Zhao and Yong Cao performed the operation and provided CCMs samples and control specimens; Jiguang Wang supervised bioinformatics studies. Jiguang Wang and Yong Cao provided overall oversight of the research.

## CONFLICT OF INTEREST STATEMENT

The authors declare no conflict of interest.

## FUNDING INFORMATION

This study is funded by Genomics Platform Construction for Chinese Major Brain Disease‐AVM (PXM2019_026280_000002‐AVM); Beijing Advanced Innovation Center for Big Data‐based Precision Medicine (PXM2020_014226_000066). Research in Wang lab is supported by RGC grants (16102522, C6021‐19EF), ITC grant (ITCPD/17‐9), a project of Hetao Shenzhen‐Hong Kong Science and Technology Innovation Cooperation Zone (HZQB‐KCZYB‐2020083), and the Padma Harilela Professorship.

## ETHICS STATEMENT

The study was approved by the Institutional Review Board and the Ethics Committee of Beijing Tiantan Hospital. (KY2017‐035‐02). The Animal Welfare and Ethics Committee of Beijing Neurosurgical Institute Laboratory approved all animal ethics and protocols.

## Supporting information

Supporting Information

Supporting Information

Supporting Information

Supporting Information

Supporting Information

Supporting Information

Supporting Information

## Data Availability

Deidentified data that are not published within this article will be made available to any qualified investigator upon request.
