# Supplementary material for: Genotype‐phenotype correlations in multiple lesions of familial cerebral cavernous malformations concerning phosphatidylinositol 3‐kinase catalytic subunit alpha mutations
Source: Clin Transl Med. 2024 Mar 7;14(3):e1610. doi: 10.1002/ctm2.1610 (PMC10918732; doi:10.1002/ctm2.1610)
Supplement: Supplementary file 1 — Supporting Information [file CTM2-14-e1610-s003.docx]

**Supplementary Figures**

**Figure S1**


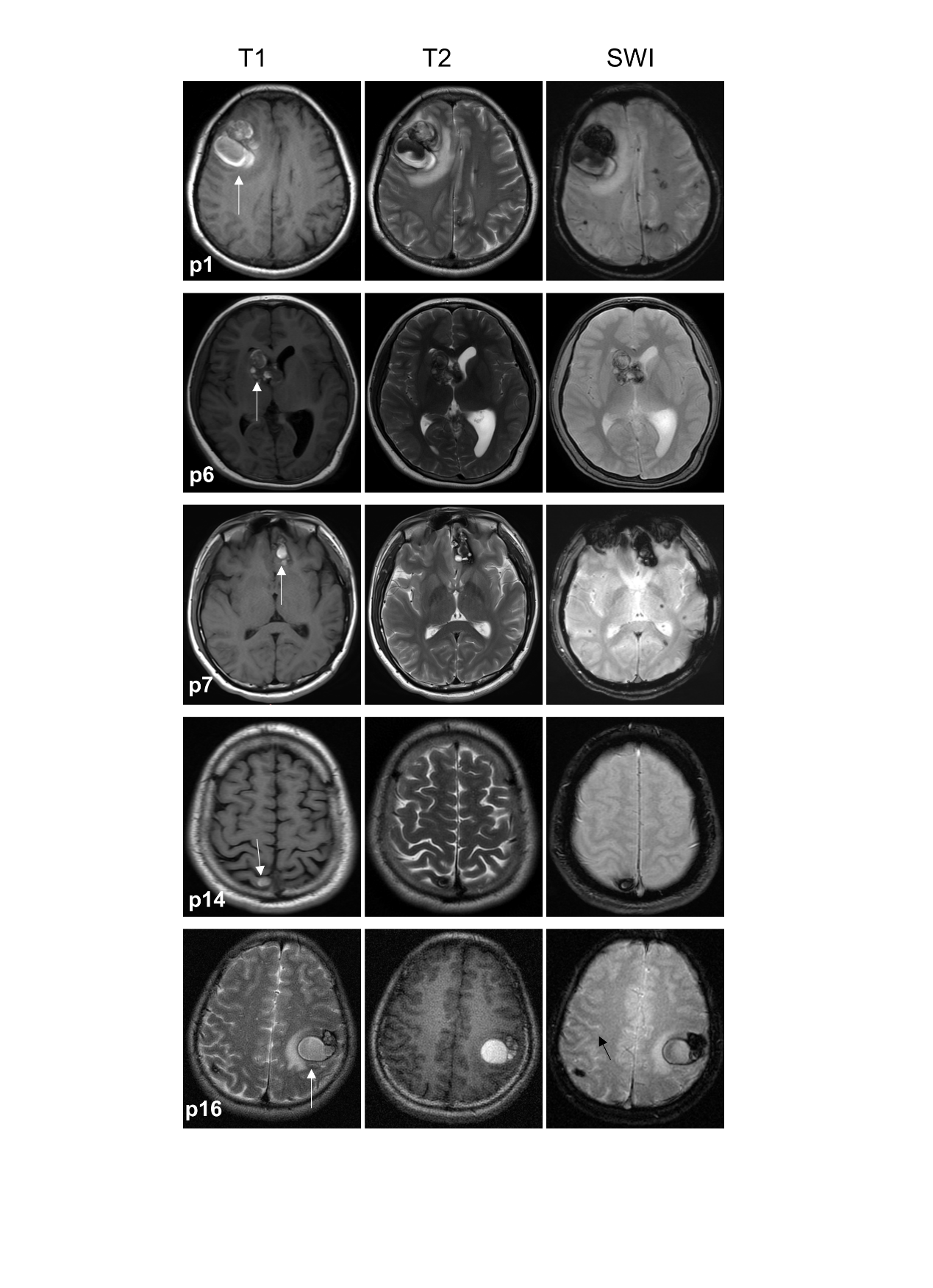


**Figure S1 continued**


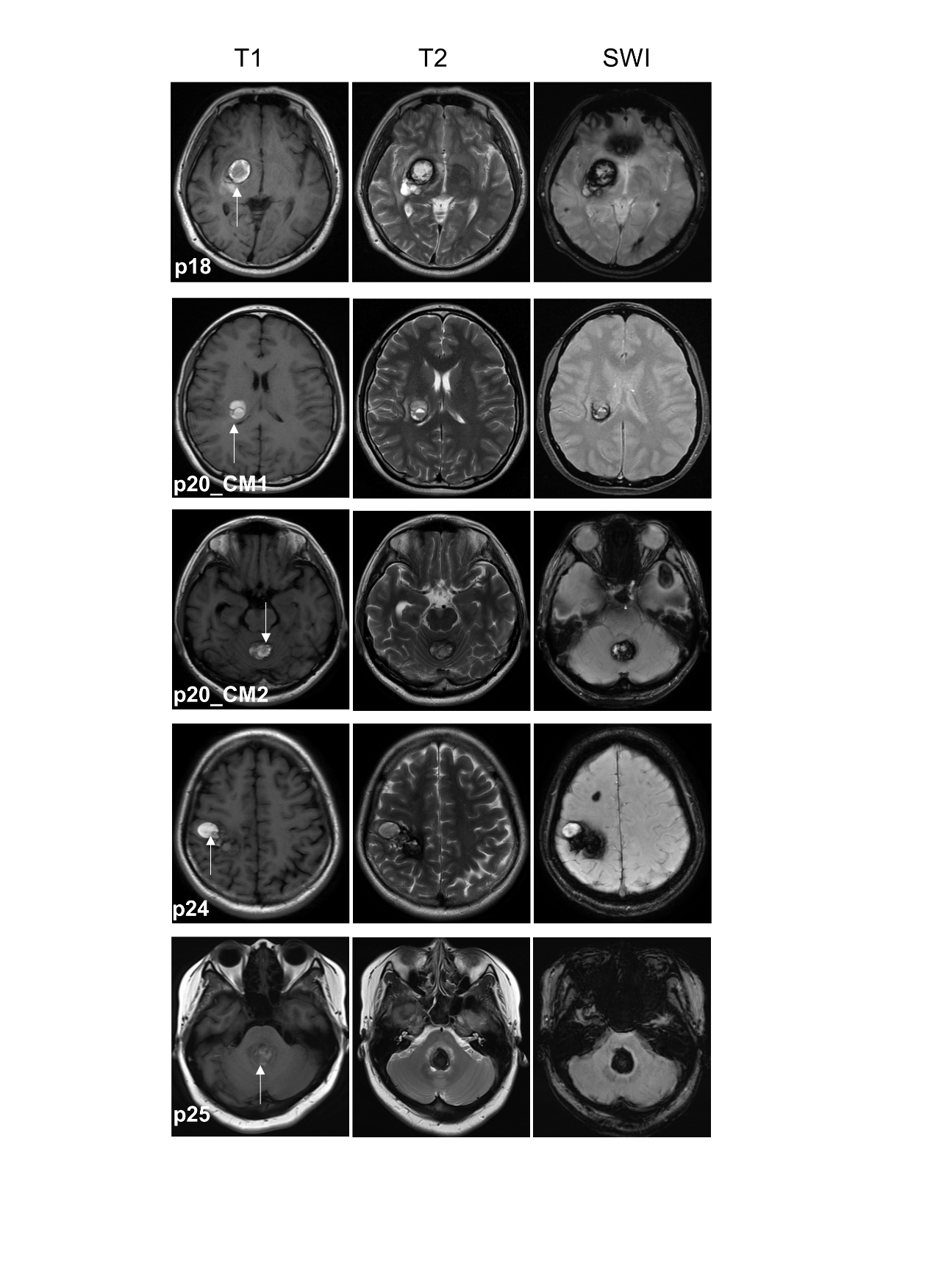


**Figure S1 continued**
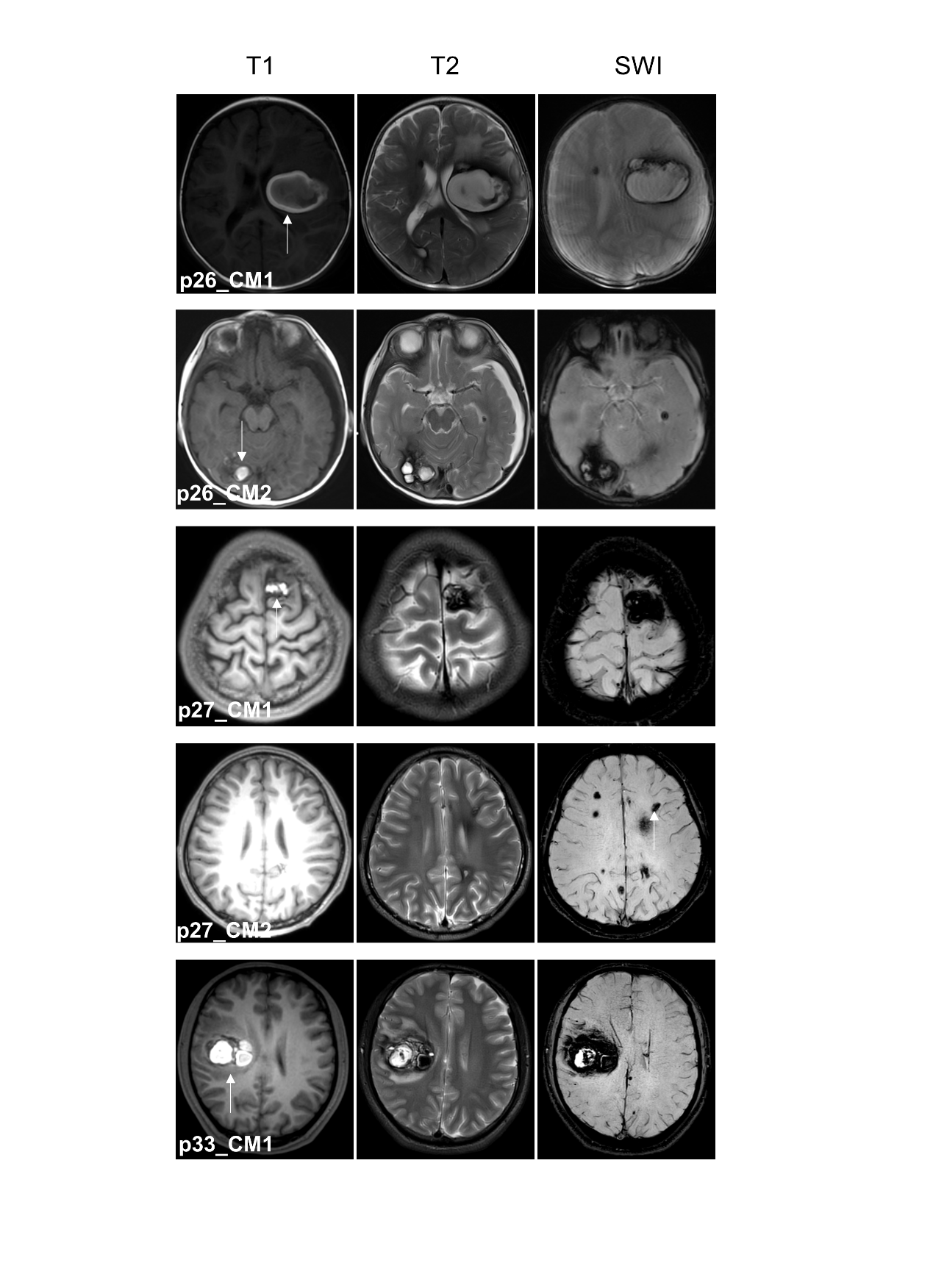


**Figure S1 continued**


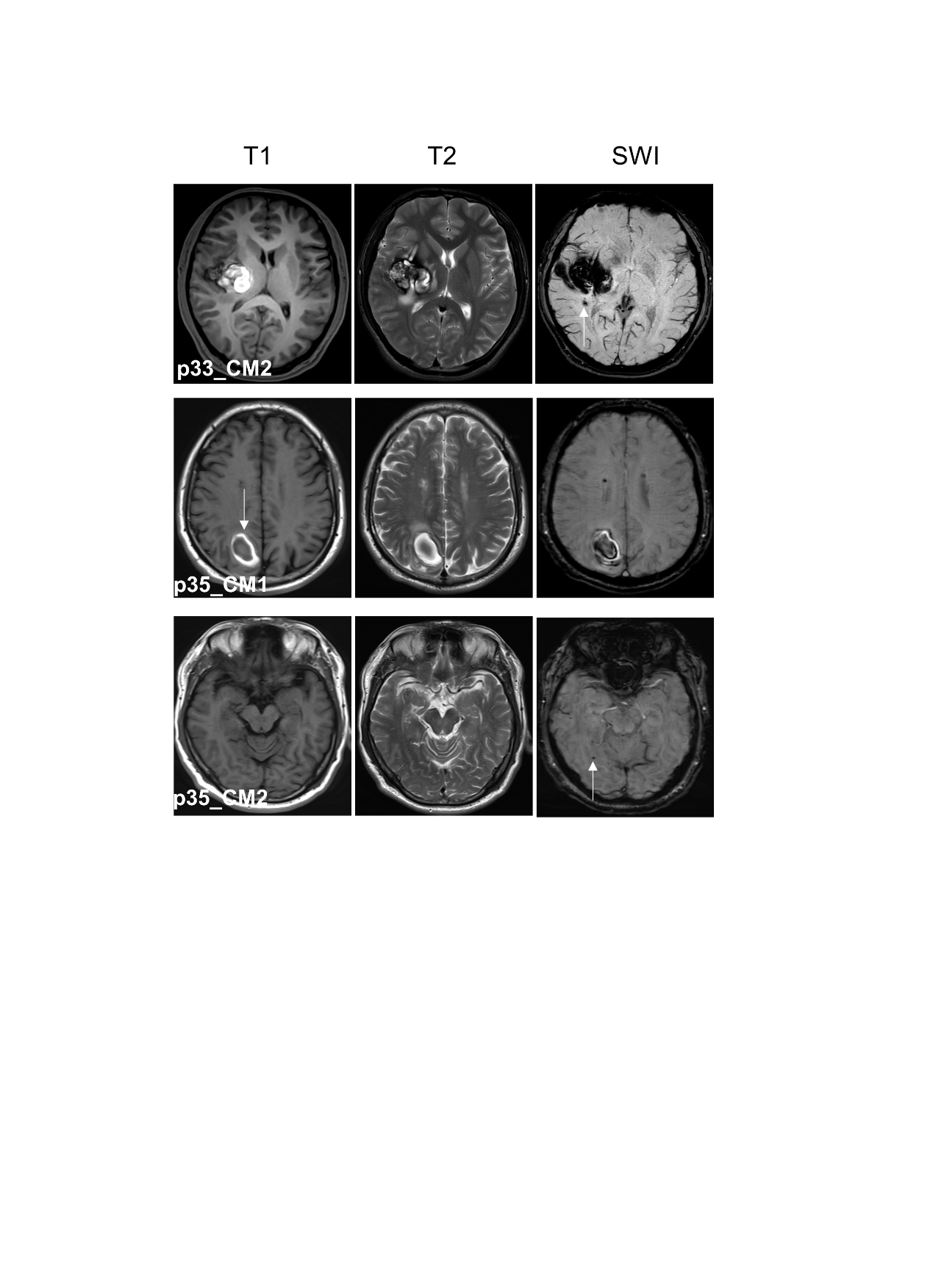


**Figure S1.** The MRI of 13 patients with familial CCMs in this study. Numbering of individuals was given at the left. p27_CM1, p27_CM2, p33_CM1, p33_CM2, p35_CM1 and p35_CM2 surgical samples were fresh frozen samples, and the rest samples were FFPE samples. White arrows (surgical samples)

Figure S2


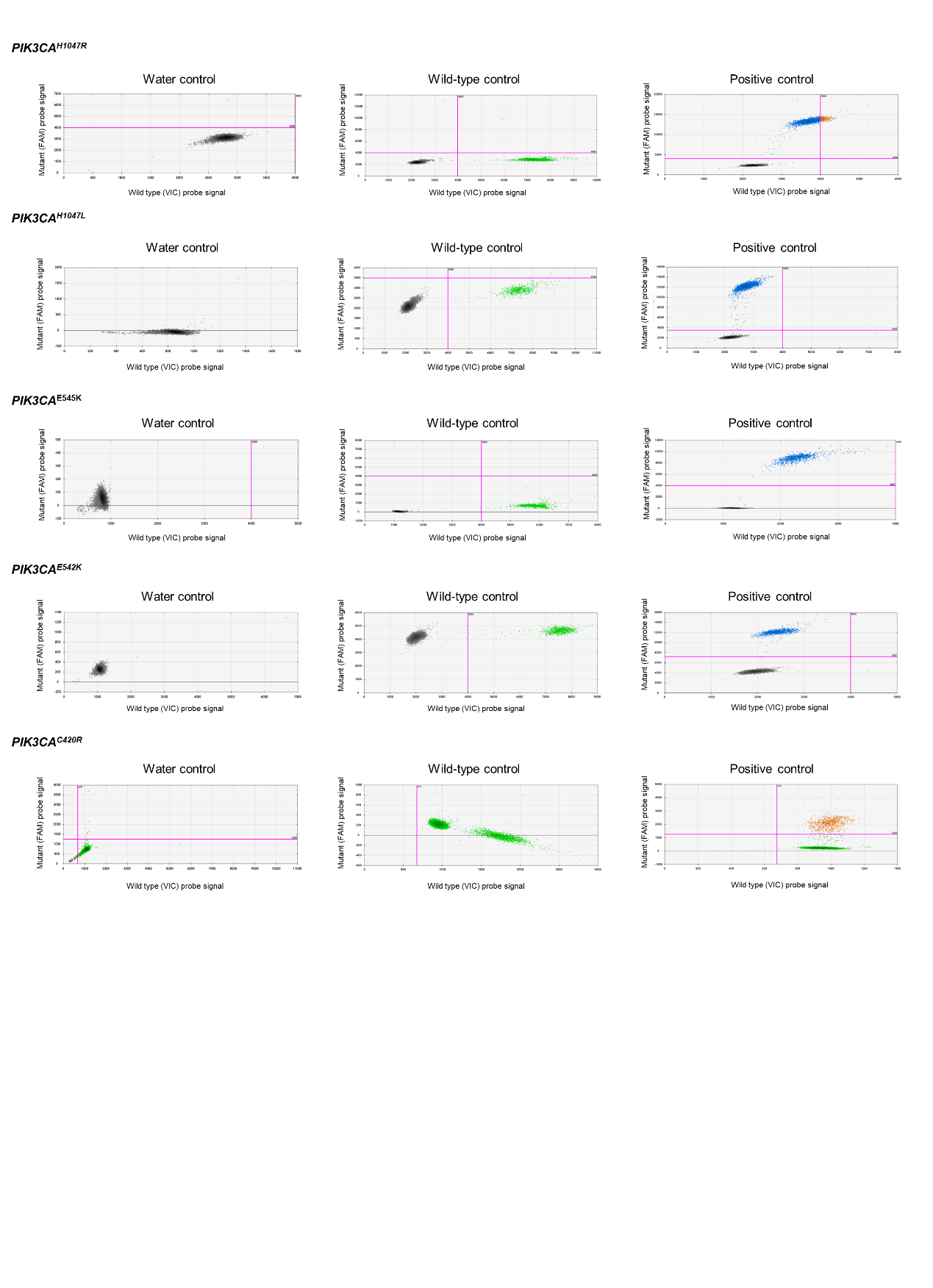


Figure S2. Water control, wild-type control and positive control for *PIK3CA^H1047R^*, *PIK3CA^H1047L^*, *PIK3CA^E545K^*, *PIK3CA^E542K^* and *PIK3CA^C420R^* mutations in the ddPCR experimental results. Dots represent single well data. Blue: the droplet encloses at least one copy of mutant template. Green: the droplet encloses at least one copy of wild-type template. Orange: the droplet encloses at least one copy of wild-type and one copy of mutant template. Black: the droplet encloses no target molecular; x axis: the signal intensities of HEX probe for the reference allele; y axis: the signal intensities of FAM probe for the mutant allele.

**Figure S3**


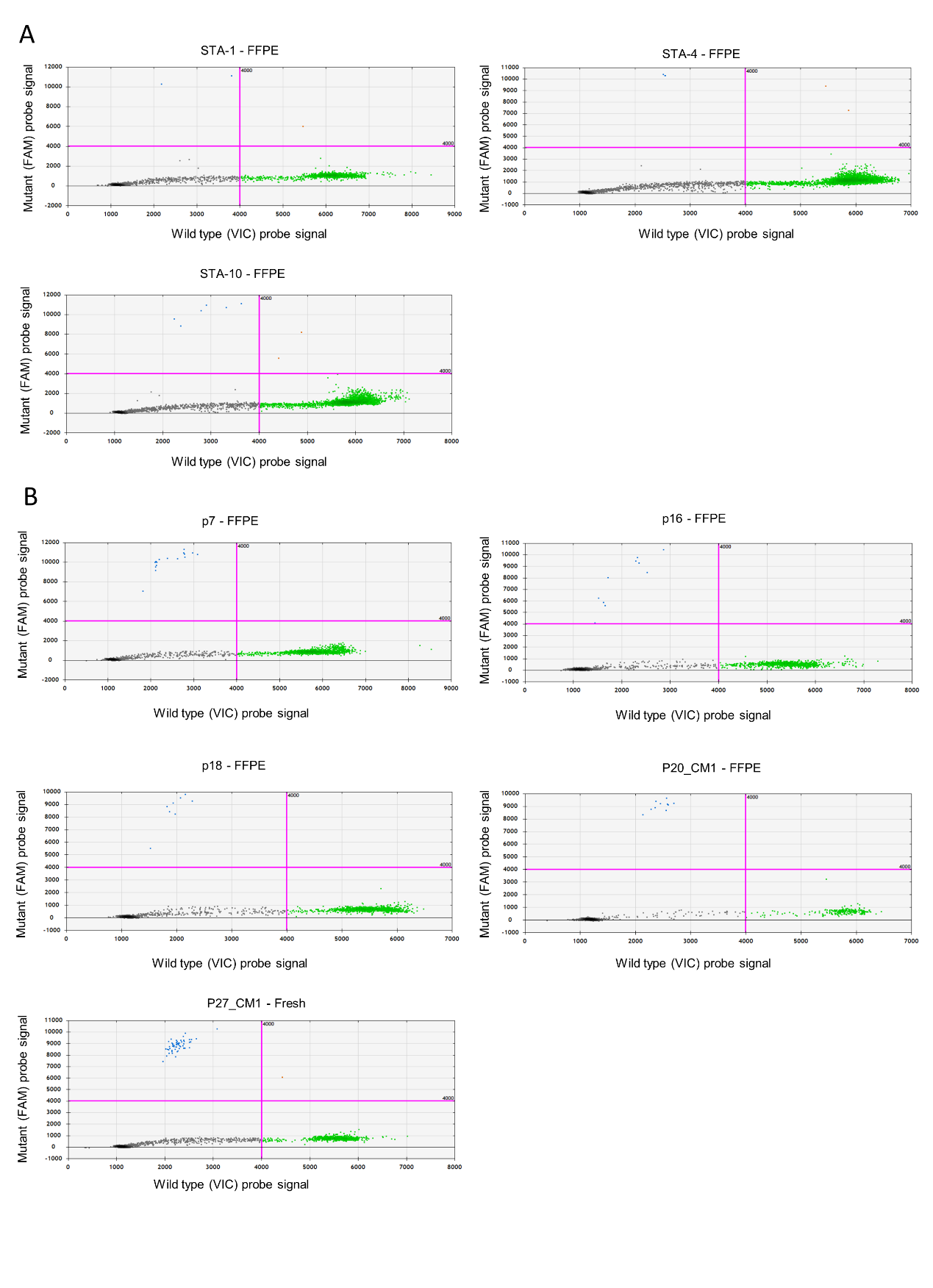


**Figure S3. Amplitude scatter plots of *PIK3CA*^E545K^ mutation ddPCRs.** A, Amplitude scatter plots of *PIK3CA*^E545K^ mutation ddPCRs of normal superficial temporal artery (STA). B, Amplitude scatter plots of *PIK3CA*^E545K^ mutation ddPCRs of mutant samples. Dots represent single well data. Blue: the droplet encloses at least one copy of mutant template. Green: the droplet encloses at least one copy of wild-type template. Orange: the droplet encloses at least one copy of wild-type and one copy of mutant template. Black: the droplet encloses no target molecular; x axis: the signal intensities of HEX probe for the reference allele; y axis: the signal intensities of FAM probe for the mutant allele.

**Figure S4**


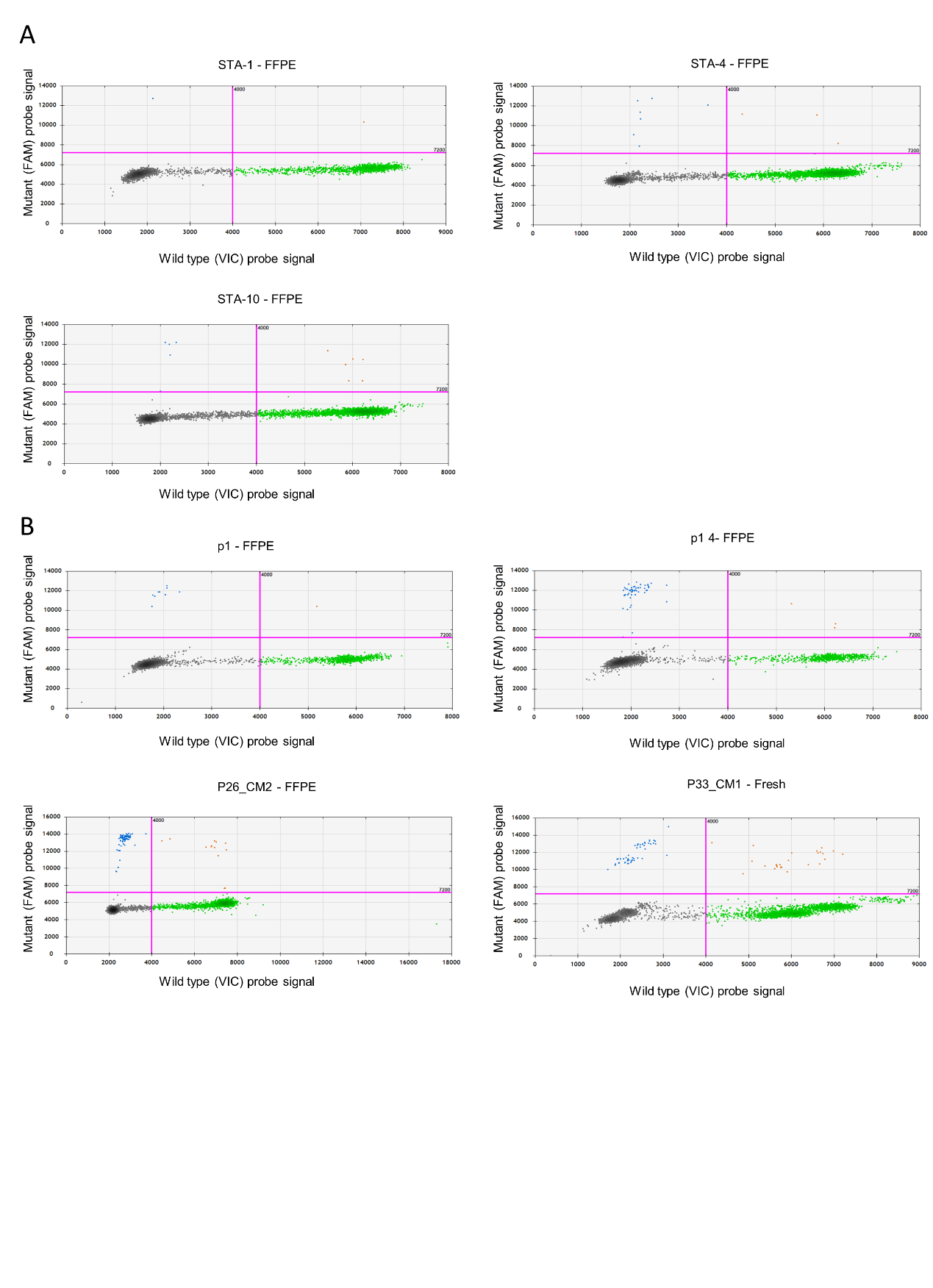


Figure S4. Amplitude scatter plots of *PIK3CA*^E542K^ mutation ddPCRs. A, Amplitude scatter plots of *PIK3CA*^E542K^ mutation ddPCRs of normal superficial temporal artery (STA). B, Amplitude scatter plots of *PIK3CA*^E542K^ mutation ddPCRs of mutant samples. Dots represent single well data. Blue: the droplet encloses at least one copy of mutant template. Green: the droplet encloses at least one copy of wild-type template. Orange: the droplet encloses at least one copy of wild-type and one copy of mutant template. Black: the droplet encloses no target molecular; x axis: the signal intensities of HEX probe for the reference allele; y axis: the signal intensities of FAM probe for the mutant allele.

**Figure S5**
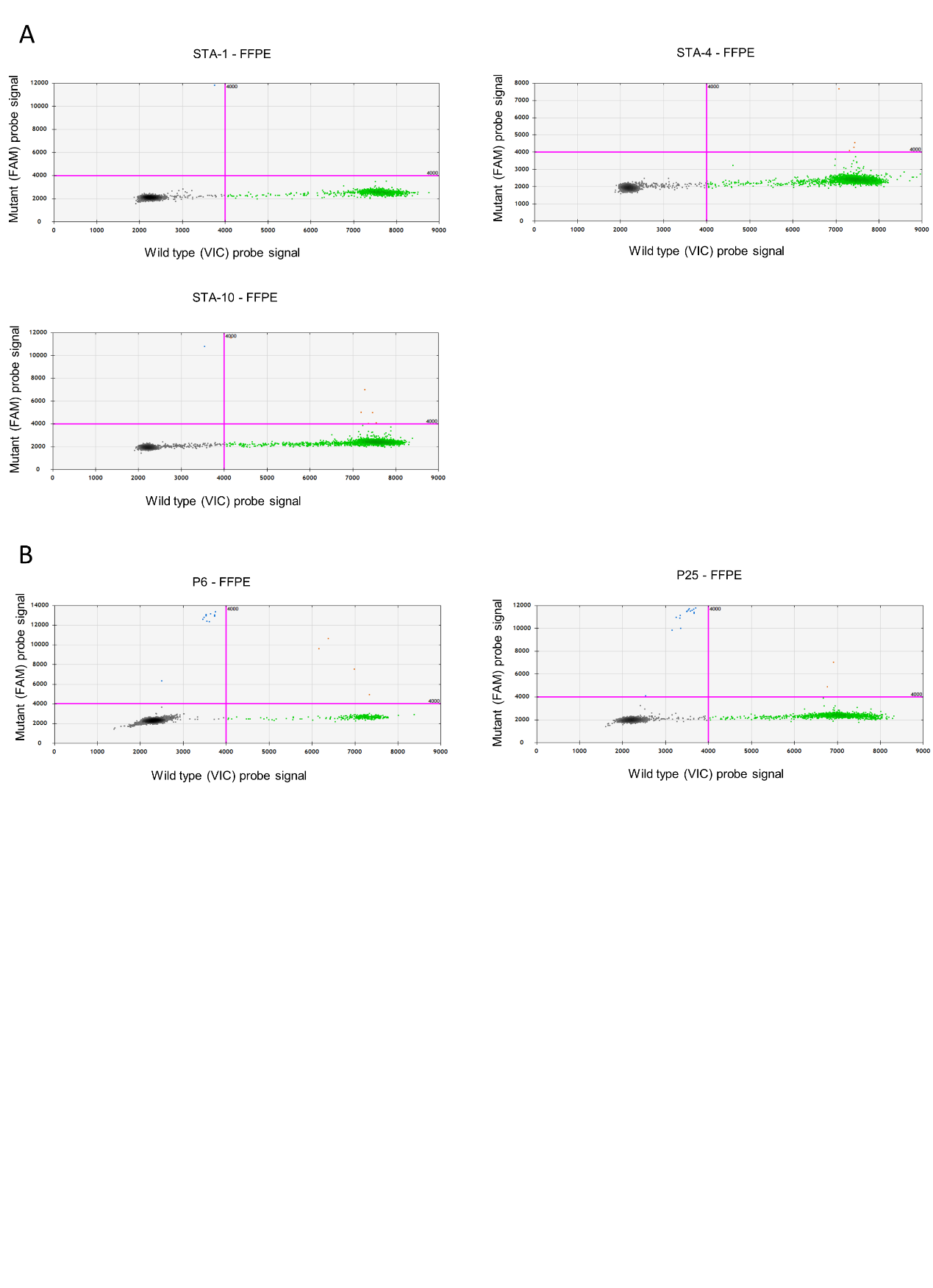


**Figure S5. Amplitude scatter plots of *PIK3CA*^H1047R^ mutation ddPCRs.** A, Amplitude scatter plots of *PIK3CA*^H1047R^ mutation ddPCRs of normal superficial temporal artery (STA). B, Amplitude scatter plots of *PIK3CA*^H1047R^ mutation ddPCRs of mutant samples. Dots represent single well data. Blue: the droplet encloses at least one copy of mutant template. Green: the droplet encloses at least one copy of wild-type template. Orange: the droplet encloses at least one copy of wild-type and one copy of mutant template. Black: the droplet encloses no target molecular; x axis: the signal intensities of HEX probe for the reference allele; y axis: the signal intensities of FAM probe for the mutant allele.

**Figure S6**
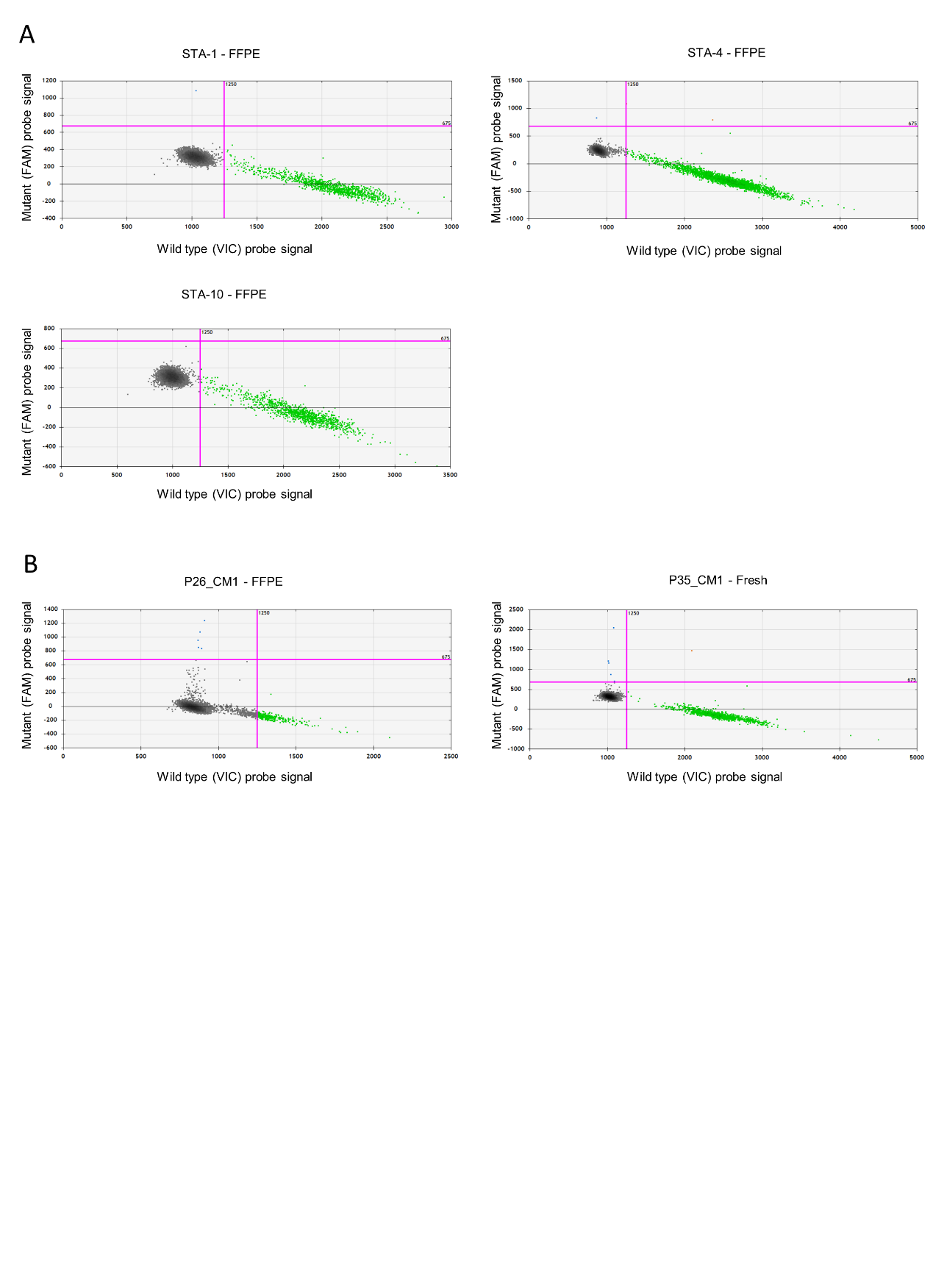


**Figure S6. Amplitude scatter plots of *PIK3CA*^C420R^ mutation ddPCRs.** A, Amplitude scatter plots of *PIK3CA*^C420R^ mutation ddPCRs of normal superficial temporal artery (STA). B, Amplitude scatter plots of *PIK3CA*^C420R^ mutation ddPCRs of mutant samples. Dots represent single well data. Blue: the droplet encloses at least one copy of mutant template. Green: the droplet encloses at least one copy of wild-type template. Orange: the droplet encloses at least one copy of wild-type and one copy of mutant template. Black: the droplet encloses no target molecular; x axis: the signal intensities of HEX probe for the reference allele; y axis: the signal intensities of FAM probe for the mutant allele.

**Figure S7**
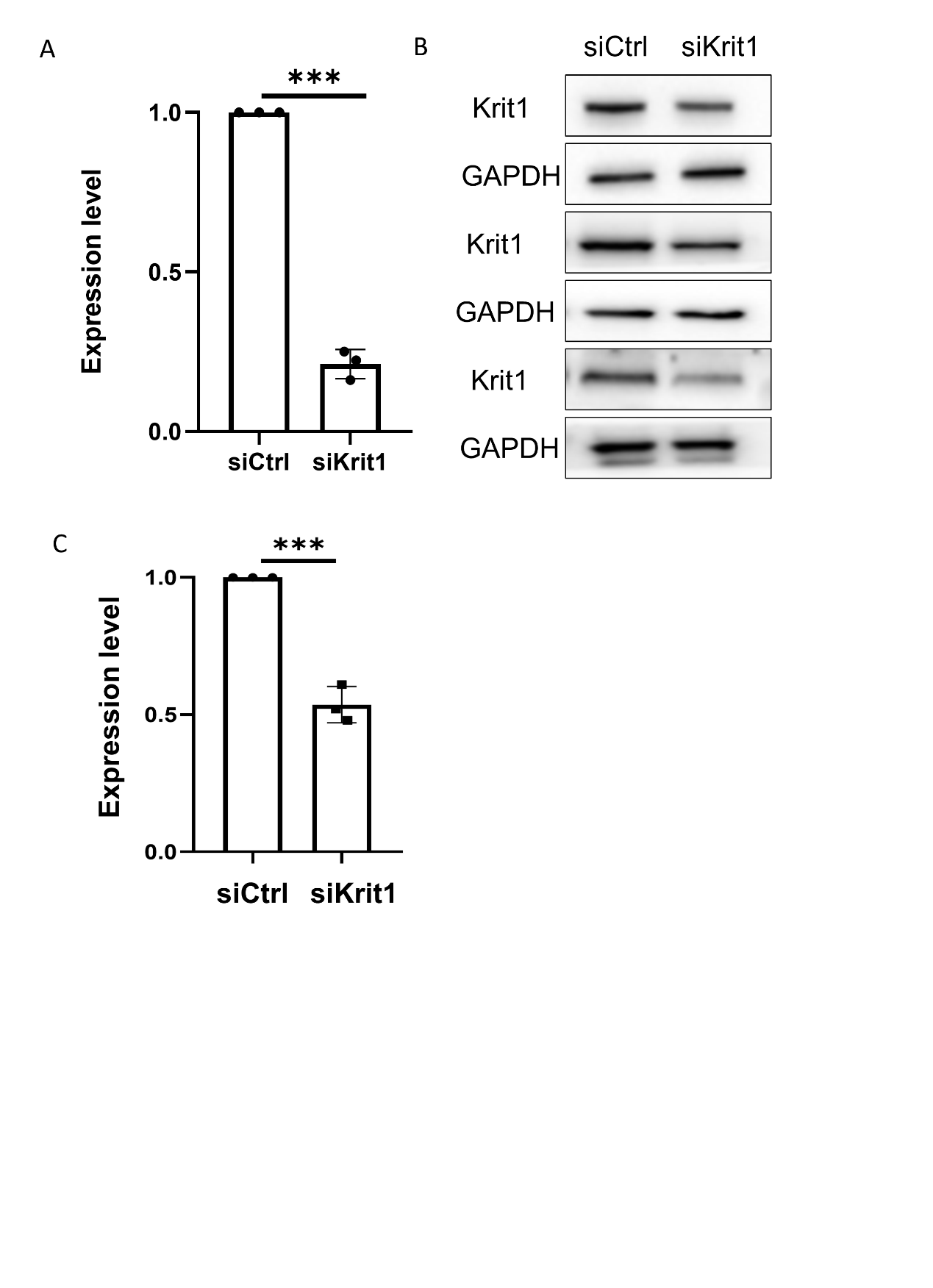


**Figure S7. Expression of Krit1 among siCtrl and siKrit1 group.** After transfected with siCtrl and siKrit1 in normal HUVECs, A, at 24h, real-time quantitative polymerase chain reaction (RT-qPCR) showed that compared to siCtrl group, the expression of Krit1 in siKrit1 group was significantly reduced (n=3 per group). B and C, at 48h, Western blot showed that compared to siCtrl group, the expression of Krit1 in siKrit1 group was significantly reduced (n=3 per group). ***p<0.001.

**Figure S8**
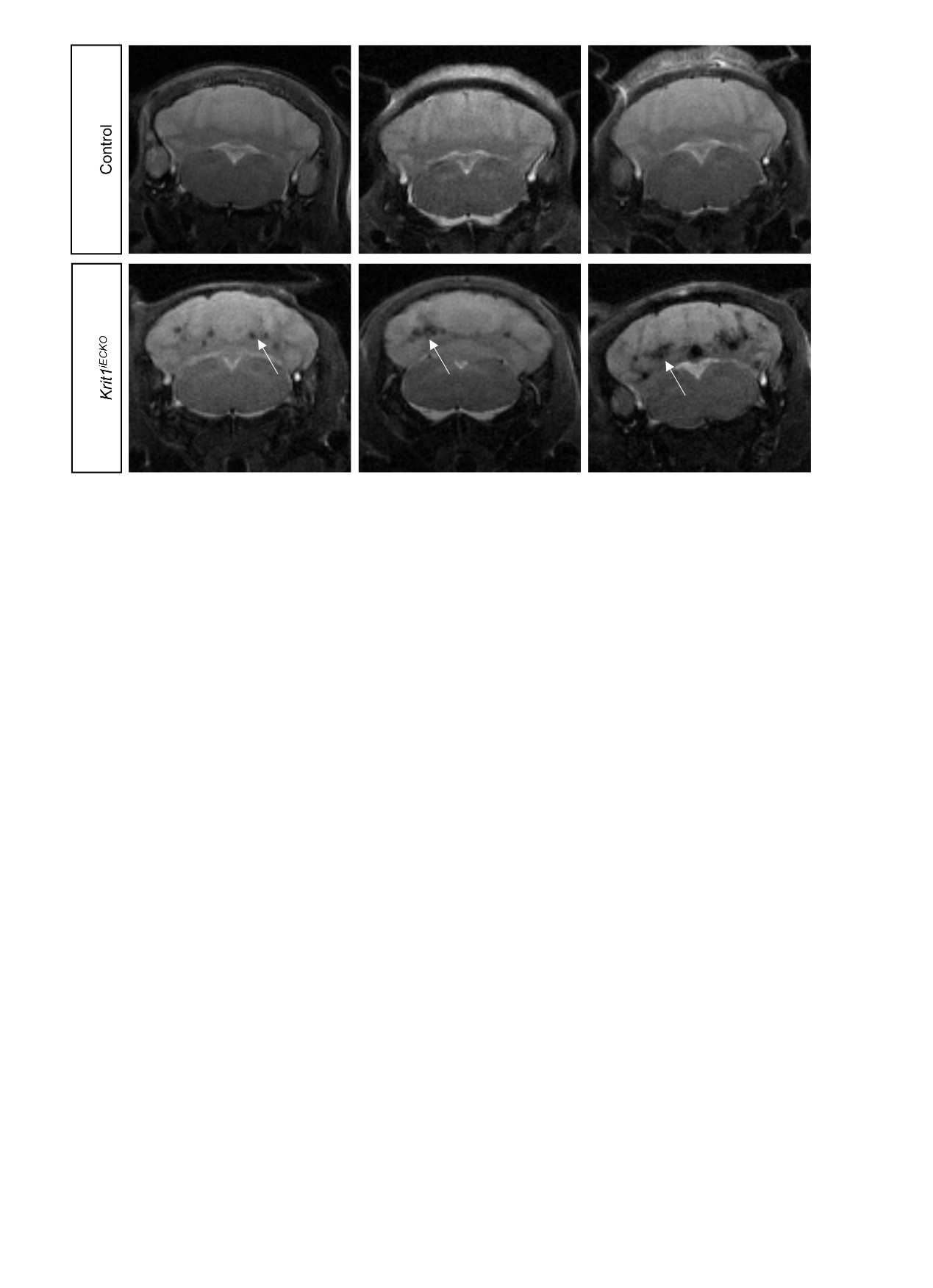


**Figure S8.** Endothelial cells deletion of krit1 induces CCM lesions in mice**.** We induced EC-specific deletion of Krit1 in Cdh5-CreERT2; Krit1^fl/fl^ (Krit1^iECKO^) mice, by 4-hydroxytamoxifen injection at P1. MRI indicates CCM lesions located in cerebellum at P30. White arrows (CCM lesions).

**Figure S9**
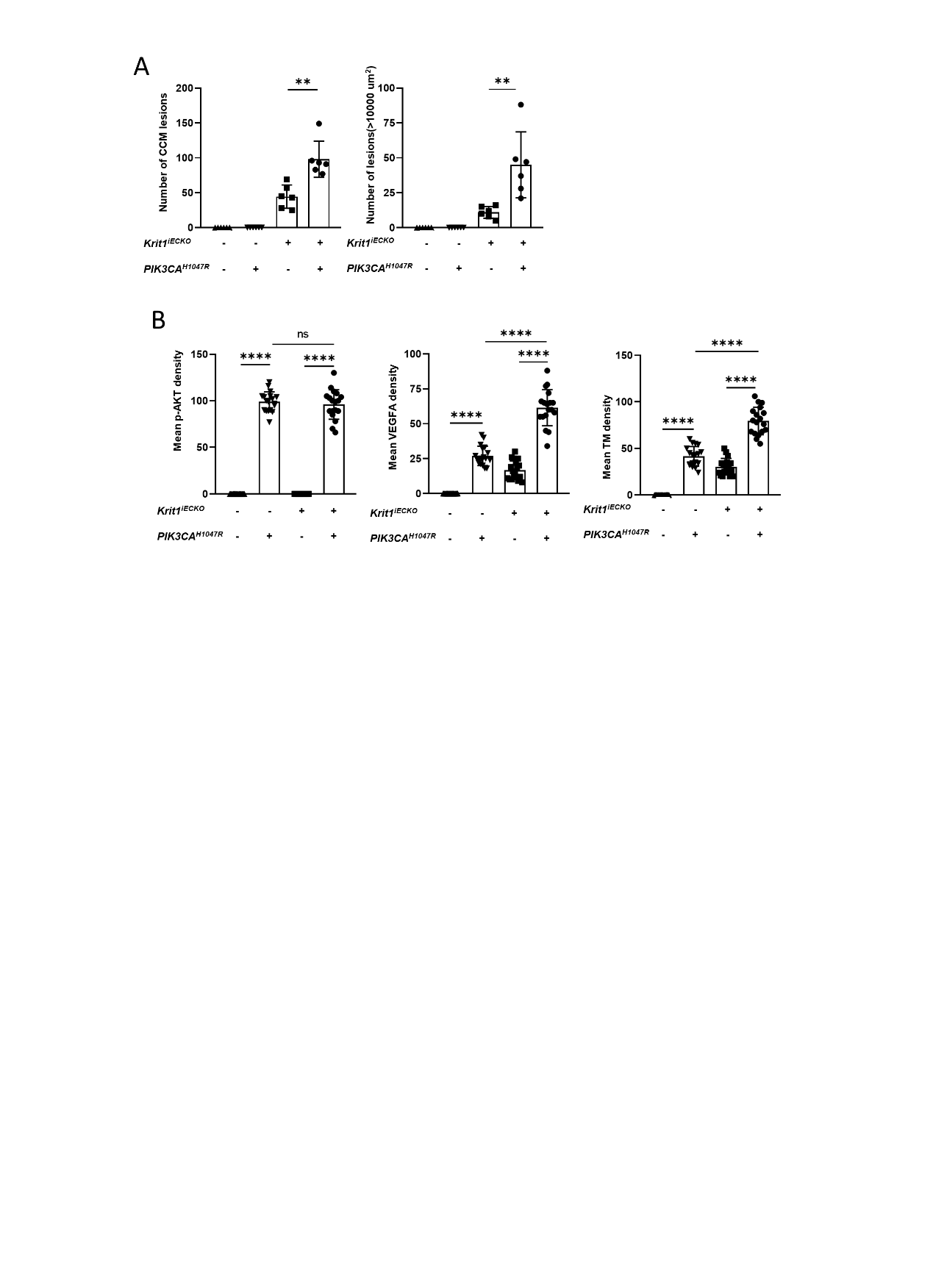


**Figure S9.** **Quantification in Figure 3.** A, the numbers of total lesions and lesions with different sizes (sized >10000 um^2^) were quantified per 10 sagittal sections, which were 200 μm apart. (CTRL: AAV-control+Krit1^fl/fl^, *PIK3CA*^H1047R^: AAV-*PIK3CA*^H1047R^+Krit1^fl/fl^, Krit1^iECKO^: AAV-control+Krit1^iECKO^, *PIK3CA*^H1047R^+Krit1^iECKO^: AAV-*PIK3CA*^H1047R^+Krit1^iECKO^, n=6 mice per group). B, Statistical analysis of mean immunofluorescence density of phosphor-AKT, VEGFA and TM among four groups (CTRL: AAV-control+Krit1^fl/fl^, *PIK3CA*^H1047R^: AAV-*PIK3CA*^H1047R^+Krit1^fl/fl^, Krit1^iECKO^: AAV-control+Krit1^iECKO^, *PIK3CA*^H1047R^+Krit1^iECKO^: AAV-*PIK3CA*^H1047R^+Krit1^iECKO^, n=6 mice per group). **, P <0.01; ****, P <0.0001.
