# Supplementary material for: Genotype‐phenotype correlations in multiple lesions of familial cerebral cavernous malformations concerning phosphatidylinositol 3‐kinase catalytic subunit alpha mutations
Source: Clin Transl Med. 2024 Mar 7;14(3):e1610. doi: 10.1002/ctm2.1610 (PMC10918732; doi:10.1002/ctm2.1610)
Supplement: Supplementary file 6 — Supporting Information [file CTM2-14-e1610-s005.docx]

**Supplementary Methods**

**Study design and patients**

This study consecutively enrolled eligible patients with familial CCMs in Beijing Tiantan Hospital between 2008 and 2021. Familial CCMs was defined as the presence of multiple CCM (typically five or more) and the presence of at least a germline mutations in one of the three genes (*CCM1/KRIT1*, *CCM2/MGC4607*, and *CCM3/PDCD10*) with or without positive family history.^1-3^ Our inclusion criteria for familial CCMs are based on a reference article in the year of 2016.^1^ Actually, in our study the patients with at least a germline mutation in one of the three genes (CCM1/KRIT1, CCM2/MGC4607, and CCM3/PDCD10) all had more than five lesions and no patient was excluded because the number of multiple lesions was less than 5. In the work of Santos and colleagues, familial CCM patients are diagnosed based on the radiographic findings: confirmed familial CCM cases are multiple CCM with at least one other member of the same family with CCM; assumed familial CCM cases are multiple CCM and either no concerned relative or unknown familial history. However, the study reported that positive genotype was found in 97% of confirmed cases and 57% of assumed cases, and there are 43% assumed cases with unknown genotype.^4^ Since our study’ concern is the genotype in the pathogenesis of familial CCM, our major inclusion criteria for familial CCM are based on the genotype, and we continued to investigate their genotype-phenotype correlations. So, we did not use Santos’ classification. Patients were included if the following inclusion criteria were met: 1) the patients harbored multiple CCMs, which presented as the multiple abnormal hypointense signals on the susceptibility weighted imaging (SWI) sequence of MRI; 2) the patients underwent at least two MRI and clinical follow-up; 3) the interval between the initial and last clinical follow-up was at least 24 months; and 4) resected ICH specimens were obtained to detected the genotype of CCMs by whole-exome sequencing (WES) and droplet digital polymerase chain reaction (ddPCR). Patients with sporadic CCMs or any prior brain irradiation were excluded. The study protocol was approved by the Ethics Committee for Human Research at the Beijing Tiantan Hospital and was in accordance with the principles of the Declaration of Helsinki (KY2017-035-02).

**Data collection and outcome**

We evaluated the demographic information and brain MRI of all the enrolled patients. Demographic information including age, sex, symptoms and familial history was carefully reviewed. According to previous studies, we classified all multiple CCMs lesions into two subtypes: ICH lesions presenting as subacute hemorrhage and causes related symptoms, while dot-sized multifocal lesions rarely cause symptom. On MRI, ICH lesions presented as hyperintense lesions on T1-weighted images (T1WI) and hyper or hypointense core on T2-weighted images (T2WI) sequences, while dot-sized lesions appeared as iso or hypointense signal on T1WI and T2WI, or poorly observed on T1WI and T2WI but punctate hypointense lesions are visualized on SWI or GRE sequence (Figure 1A). The type of multiple CCM lesions identified by initial MRI scans was independently determined by 3 neurosurgeons (J.W., Y.M.J. and R.H.). Discrepancies were resolved by a senior neurosurgeon (Y.C.). All enrolled patients with multiple CCMs both performed MRI and clinical follow-up in our hospital. The primary clinical outcome was the occurrence of a hemorrhage event. Hemorrhage event was defined as a symptomatic event with radiographic evidence of overt intracerebral hemorrhage.^5, 6^ The hemorrhagic events were assessed prior to resection of the lesion.

**Sample preparation**

Pairings of symptomatic ICH lesions and their adjacent independent dot-sized lesions, which were near from or in the surgical route in reaching ICH lesions were obtained by micro-surgically resection (Supplementary Movie 1). Operative CCM specimens and paired peripheral whole blood samples were obtained from enrolled patients for further WES. For fresh frozen samples (N=6), specimens were preserved in liquid nitrogen within 5 minutes after resection, while blood samples (N=3) were stored in -80 °C refrigerator. FFPE samples (N=12) were obtained from the CCM tissue bank at the Department of Neurosurgery at Beijing Tiantan Hospital. Overall, the 15 ICH lesions consisted of 3 fresh-frozen operative ICH specimens from 3 patients and 12 FFPE tissue surgical ICH samples from another 10 patients. All patients signed an informed consent agreement and the study was approved by the Institutional Review Board and the Ethics Committee of Beijing Tiantan Hospital.

**DNA isolation from frozen and FFPE samples**

For frozen CCMs specimens and paired peripheral blood samples, commercially available kits (QIAGEN Gentra Puregene and QIAamp DNA Blood Mini Kit) were used following manufacturer’s recommendations. For FFPE samples, genomic DNA was extracted by QIAamp GeneRead DNA FFPE kits (Qiagen) following manufacturer’s recommendations and the Uracil-N-Glycosilase (UNG) were used to remove artificially induced uracils from the DNA obtained from the FFPE sample.

**Whole exome sequencing (WES) of CCM samples and peripheral blood samples**

WES of CCM specimens and paired peripheral blood samples were performed as our previous study.^7, 8^ The quality of isolated genomic DNA was verified by: (1) DNA degradation and contamination were monitored on 1% agarose gels; (2) DNA concentration was measured by Qubit® DNA Assay Kit in Qubit® 2.0 Fluorometer (Invitrogen, USA). For library preparation, a total amount of 0.6 μg genomic DNA per sample was used as input material. Sequencing libraries were generated using Agilent SureSelect Human All Exon V6 kit (Agilent Technologies, CA, USA) following manufacturer’s recommendations and index codes were added to each sample. Fragmentation was carried out by the hydrodynamic shearing system (Covaris, Massachusetts, USA) to generate 180-280 bp fragments. Remaining overhangs were converted into blunt ends via exonuclease/polymerase activities. After adenylation of 3’ ends of DNA fragments, adapter oligonucleotides were ligated. DNA fragments with ligated adapter molecules on both ends were selectively enriched in a PCR reaction. After PCR reaction, libraries were hybridized in liquid phase with the biotin-labeled probes and captured using magnetic beads enriched for the exons of genes. Captured libraries were enriched in a PCR reaction to add index tags to prepare for sequencing. Products were purified using AMPure XP system (Beckman Coulter, Beverly, USA) and quantified using the Agilent high sensitivity DNA assay on the Agilent Bioanalyzer 2100 system. The clustering of the index-coded samples was performed on a cBot Cluster Generation System using Hiseq PE Cluster Kit (Illumina) according to the manufacturer’s instructions. After cluster generation, the DNA libraries were sequenced on Illumina Hiseq platform and 150 bp paired-end reads were generated. The depth of CCM lesions and blood controls was 300X and 100X, respectively.

**Droplet digital polymerase-chain-reaction (ddPCR)**

All CCM samples were performed ddPCR to investigate the *PIK3CA* variants in Shanghai Biotechnology Corporation. To detect the *PIK3CA*^E542K^, *PIK3CA*^E545K^, *PIK3CA*^H1047R^ and *PIK3CA*^C420R^ in CCM samples, template DNA were analyzed on the QX200 Droplet Digital PCR system (Bio-Rad Laboratories, USA). The ddPCR probes were synthesized by Shanghai BiOligo Biotech Co., Ltd (Shanghai, China). As described in our previous study, a total of 200 ul 40X probe assay containing 60 ul forward primer (100 uM), 60 ul reverse primer (100 uM), 20 ul reference probe (100 uM), 20 ul mutant allele probe (100 uM), and 40 ul DNase/RNase free water was prepared. The 20 µl reaction mix consisted of 10 µl of 2x ddPCR SuperMix for probes (Bio-Rad Laboratories, USA), 0.5 µl of the 40X probe assay, 7.5 µl DNase/RNase free water and 2 µl of 5 ng/µl genomic DNA. Cycling conditions for the reaction were 95°C for 10 min, followed by 40 cycles of 94°C for 30 sec and 60°C for 1 min, then 98°C for 10 minutes, and finally a 16°C hold on a Life Technologies Veriti thermal cycler. All assays were validated by temperature gradient to ensure optimal separation of reference and variant signals. Data were analyzed using QuantaSoft v1.7.4 (Bio-Rad Laboratories, USA). The 300 bp synthetic mutant DNA fragment (*PIK3CA*^E542K^ mutation [*PIK3CA*-alt], *PIK3CA*^E545K^, mutation [*PIK3CA*-alt], *PIK3CA*^H1047R^ mutation [*PIK3CA*-alt] or *PIK3CA*^C420R^ mutation [*PIK3CA*-alt]) and 300 bp synthetic wildtype DNA fragment (human genomic DNA [*PIK3CA*-ref]) were constructed by Shanghai Biotechnology Corporation and used as positive control and negative control, respectively. In addition, DNase/RNase free water was used as no template control. We also performed ddPCR for normal control tissue (superficial temporal artery) from 3 FFPE individuals who were undergoing standard craniotomy procedure. To reduce false positives, only mutations supported by >5 positive droplets and >0.5% fractional abundance was reported.^9, 10^ For CCM gene germline mutations, the results of WES by 300-fold coverage demonstrates high precision and the MAF of CCM gene germline mutations was high, therefore, we did not validate the results of WES by ddPCR. Moreover, future bulk transcriptomics studies are helpful for understanding the role of molecular mechanisms in genotype-phenotype correlations in of familial cerebral cavernous malformations.^11-14^

**Culture and treatment of human umbilical vein endothelial cells (HUVECs)**

Primary HUVECs were purchased from ScienCell and cultured in endothelial cell medium (#1001, ScienCell) supplemented with 5% fetal bovine serum (#1001, ScienCell), 1% endothelial cell growth supplement (#1001, ScienCell), and 1% penicillin/streptomycin (#1001, ScienCell). The cells were cultivated in a humidified atmosphere of 5% CO2 at 37 ℃. HUVECs within passage 10 were utilized in the experiments. MK-2206 2HCl (S1078, Selleck), an inhibitor of AKT signaling and Fasudil (S1573, Selleck), an inhibitor of ROCK signaling was added into complete medium directly.

**Short interfering RNA (siRNA) transfection**

HUVECs were transfected with siRNA directed against *PTEN* or *Krit1* (Beijing SyngenTech Co., LTD.) using Lipofectamine 3000 (L3000015, Invitrogen) according to the manufacturer’s protocol, with siNC (negative control) used in control groups. Western blotting was used to access the transfection efficiency. The siRNA was synthesized with the following sequences: si*PTEN*, 5′- AGCUAAAGGUGAAGAUAUATT-3′, si*Krit1*, 5′- CCAGAAACGGAUAGACAUATT-3′ and the negative control, 5′- UUCUCCGAA CGUGUCACGUTT-3′.

**RNA isolation and RT-qPCR**

24 hours after siRNA transfection or adenovirus infection, total RNA was isolated using the TRIzol reagent (Invitrogen). RNA was cleaned by using gDNA Eraser (Takara, Kyoto, Japan). After measuring the RNA concentration, purified RNA was reverse transcribed using a PrimeScript™ RT reagent Kit (Takara). QPCR was performed using TB Green Premix Ex Taq (Takara) with a QuantStudio™ 3 System (Applied Biosystems) with specific primers. GAPDH was used as a reference gene. All amplification reactions were carried out over 40 cycles (a hold stage of 30s at 95°C, then a two-step program of 3s at 95°C, 34s at 60°C). The mRNA expression for transcripts was calculated by the ΔΔCt method. The primer sequences were as follows:

*Krit1*, forward, CCATCGTACCTGTTAC; reverse, ACTGACACCTTCACTTGTACTG; GAPDH, forward, AATGACCCCTTCATTGAC; reverse, TCCACGACGTACTCAGCGC.

**Western blot**

Whole-cell lysates were prepared using RIPA buffer (89901, Thermo). Protein concentration was determined using a BCA Protein Assay kit (P0012, Beyotime). Equal amounts of total protein (20 μg) were loaded for western blot. The primary antibodies used were as follows: anti-phospho-AKT (1:1000, 4060; CST), anti-Thrombomodulin (1:1000, ab109189, abcam), anti-VEGFA (1:1000, ab46154; abcam), anti-phospho-ROCK2 (1:1000, ab228008, abcam), anti-*Krit1* (1:1000, ab196025; abcam) and anti-GAPDH (1:2500, ab9485; abcam). Secondary antibody was Goat Anti-Mouse IgG-HRP (1:1000, #7076; CST) or Goat AntiRabbit IgG-HRP (1:1000, #7074; CST). For the inhibitor assay, HUVECs were treated with S1078, S1573 or 0.1% DMSO for 24 h.

**Animals**

The Animal Welfare and Ethics Committee of Beijing Neurosurgical Institute Laboratory approved all animal ethics and protocols. All experiments were conducted under the guidelines/regulations of Capital Medical University. Endothelial-specific conditional *Krit1*-null mice (*Krit1*^iECKO^) were generated by crossing a Cdh5 promoter-driven tamoxifen-regulated Cre recombinase (Cdh5-CreERT2) with loxP-flanked *Krit1*(*Krit1*^fl/fl^). The *Krit1*^fl/fl^ and *Krit1*^iECKO^ mice were kindly donated by Professor Xiangjian Zheng (Department of Pharmacology, Tianjin Medical University, China). As previous study reported, to induce knockdown of *Krit1* deletion, 50 µL of 4-hydroxytamoxifen (0.5 mg/mL, H7904, Sigma-Aldrich) was given intragastrically to neonatal pups at postnatal day 1 (P1),^15^ then the CCM mouse model were confirmed at 4 weeks by 7.0 T small animal MRI scanner (Bruker, Germany). Litters were randomly divided into different groups for the subsequent experiments.

A**deno‑associated virus injection**

AAV-BR1-CAG-eGFP-WPRE (AAV-control) and AAV-BR1-*PIK3CA*^H1047R^-eGFP-WPRE (AAV-*PIK3CA*^H1047R^) were obtained from Genechem Co., Ltd, Shanghai, China. The P1 mice was given 4-hydroxytamoxifen and performed 2-months follow-up by MRI, then 2-month-old *Krit1*^fl/fl^ and *Krit1*^iECKO^ mice were administered 100 μl of phosphate-buffered saline (PBS) containing 1 × 10^11^ genome copies (GC) AAV-control or 1 × 10^11^ GC AAV-*PIK3CA*^H1047R^ by retro-orbital venous sinus injection as described previously.^7, 16^

**Magnetic resonance imaging (MRI)**

7.0 T small animal MRI scanner (Bruker, Germany) was performed in mice. T2WI and SWI were performed to evaluate the CCM lesions. Mice were anesthetized for imaging by airway anesthesia of isoflurane. T2-weighted images were acquired with the following parameters: repetition time (TR)=3200 ms, echo time (TE)=40 ms, field of view (FOV)=25 × 25mm, matrix=256 × 192, slices=25, and slices thickness=0.5mm. SWI was performed with the following parameters: TR=600ms, TE=8ms, FOV=25 × 25 mm2, matrix=256 × 192, slices=25, and slices thickness=0.5mm. The ICH in mice was defined as lesions with hyper or hypointense core with surrounding hypointense rim on T2WI and hypointense on SWI.

**Haematoxylin and eosin (H&E) and Immunofluorescence staining**

Mice were anesthetized with IP injection of sodium pentobarbital followed by intracardial perfusion with 20 mL of cold PBS and 4% (w/v) paraformaldehyde (PFA) in PBS (pH 7.4). The brains were carefully dissected. H&E staining was performed using a HE staining Kit (G1120, Solarbio) according to the manufacturer’s instructions. Based on previous study, the numbers of total lesions and lesions with different sizes were quantified per 10 sagittal sections, which were 200 μm apart.^17^ For immunofluorescence staining, the Opal Multiplex IHC Assay Kit (PerkinElmer, USA) was used for the paraffin embedded tissue sections according to the manufacturer’s instructions. Images were acquired on LSM 880 (Zeiss, Germany) or LSM 710 (Zeiss, Germany). Primary antibodies were: anti-CD31 (1:200, 77699, CST), anti-phospho-AKT (1:100, 4060, CST), anti-Thrombomodulin (1:500, ab230010; abcam), anti-VEGFA (1:500, ab1316, abcam) and DAPI (1:1000, C0065, Solarbio). Mean fluorescence intensity was quantified by NIH Image J software.

**Bevacizumab treatment**

Bevacizumab (A2006, Selleck) were used to suppress VEGF signaling pathway. According to previous study,^18^ bevacizumab was dissolved in DMSO for a 5mg/ml stock solution and diluted in sterile PBS for a 1mg/ml working solution. Two-month-old *Krit1*^iECKO^ mice was injected AAV-*PIK3CA*^H1047R^ by retro-orbital venous sinus, meanwhile the mice were injected through tail vein with either bevacizumab with a dose of 2 mg/kg or vehicle twice a week for total three weeks and then the mice were performed MRI and harvested at P90.

**Statistical analysis**

Standard statistical tests (Student t test, One-Way ANOVA, Fisher exact test, Pearson χ2 test, log-rank test) were performed when applicable. The incidence rates for the hemorrhage events were calculated by dividing the number of events by person-years at risk, with 95% confidence intervals estimated using a Poisson model. The cumulative incidence of hemorrhage events was presented using Kaplan-Meier curves. P values under the level of 0.05 were considered statistical significance. All statistical analyses were performed with GraphPad Prism version 8.00.

**References**

1. Mespreuve M, Vanhoenacker F, Lemmerling M. Familial multiple cavernous malformation syndrome: Mr features in this uncommon but silent threat. *J Belg Soc Radiol*. 2016;100:51

2. Verlaan DJ, Davenport WJ, Stefan H, Sure U, Siegel AM, Rouleau GA. Cerebral cavernous malformations: Mutations in krit1. *Neurology*. 2002;58:853-857

3. Denier C, Labauge P, Brunereau L, Cavé-Riant F, Marchelli F, Arnoult M, et al. Clinical features of cerebral cavernous malformations patients with krit1 mutations. *Ann Neurol*. 2004;55:213-220

4. Santos AN, Rauschenbach L, Saban D, Chen B, Darkwah Oppong M, Herten A, et al. Multiple cerebral cavernous malformations: Clinical course of confirmed, assumed and non-familial disease. *Eur J Neurol*. 2022

5. Jeon JS, Kim JE, Chung YS, Oh S, Ahn JH, Cho WS, et al. A risk factor analysis of prospective symptomatic haemorrhage in adult patients with cerebral cavernous malformation. *J Neurol Neurosurg Psychiatry*. 2014;85:1366-1370

6. Wang J, Yu QF, Huo R, Sun YF, Jiao YM, Xu HY, et al. Zabramski classification in predicting the occurrence of symptomatic intracerebral hemorrhage in sporadic cerebral cavernous malformations. *Journal of neurosurgery*. 2023:1-8

7. Huo R, Yang Y, Sun Y, Zhou Q, Zhao S, Mo Z, et al. Endothelial hyperactivation of mutant map3k3 induces cerebral cavernous malformation enhanced by pik3ca gof mutation. *Angiogenesis*. 2023;26:295-312

8. Weng J, Yang Y, Song D, Huo R, Li H, Chen Y, et al. Somatic map3k3 mutation defines a subclass of cerebral cavernous malformation. *Am J Hum Genet*. 2021;108:942-950

9. Huo R, Yang Y, Xu H, Zhao S, Song D, Weng J, et al. Somatic gja4 mutation in intracranial extra-axial cavernous hemangiomas. *Stroke Vasc Neurol*. 2023

10. Hong T, Xiao X, Ren J, Cui B, Zong Y, Zou J, et al. Somatic map3k3 and pik3ca mutations in sporadic cerebral and spinal cord cavernous malformations. *Brain*. 2021;144:2648-2658

11. Scimone C, Granata F, Longo M, Mormina E, Turiaco C, Caragliano AA, et al. Germline mutation enrichment in pathways controlling endothelial cell homeostasis in patients with brain arteriovenous malformation: Implication for molecular diagnosis. *International journal of molecular sciences*. 2020;21

12. Scimone C, Donato L, Alafaci C, Granata F, Rinaldi C, Longo M, et al. High-throughput sequencing to detect novel likely gene-disrupting variants in pathogenesis of sporadic brain arteriovenous malformations. *Frontiers in genetics*. 2020;11:146

13. Donato L, Alibrandi S, Scimone C, Rinaldi C, Dascola A, Calamuneri A, et al. The impact of modifier genes on cone-rod dystrophy heterogeneity: An explorative familial pilot study and a hypothesis on neurotransmission impairment. *PloS one*. 2022;17:e0278857

14. Scimone C, Bramanti P, Ruggeri A, Katsarou Z, Donato L, Sidoti A, et al. Detection of novel mutation in ccm3 causes familial cerebral cavernous malformations. *J Mol Neurosci*. 2015;57:400-403

15. Choi JP, Yang X, He S, Song R, Xu ZR, Foley M, et al. Ccm2l (cerebral cavernous malformation 2 like) deletion aggravates cerebral cavernous malformation through map3k3-klf signaling pathway. *Stroke*. 2021;52:1428-1436

16. Park ES, Kim S, Huang S, Yoo JY, Korbelin J, Lee TJ, et al. Selective endothelial hyperactivation of oncogenic kras induces brain arteriovenous malformations in mice. *Ann Neurol*. 2021;89:926-941

17. Zhou HJ, Qin L, Jiang Q, Murray KN, Zhang H, Li B, et al. Caveolae-mediated tie2 signaling contributes to ccm pathogenesis in a brain endothelial cell-specific pdcd10-deficient mouse model. *Nat Commun*. 2021;12:504

18. Xu M, Xu HH, Lin Y, Sun X, Wang LJ, Fang ZP, et al. Lect2, a ligand for tie1, plays a crucial role in liver fibrogenesis. *Cell*. 2019;178:1478-1492 e1420
